# Effectiveness of Gatekeeper Training Program (GTP) on awareness, attitude, mental help seeking intention and gatekeeper behavior among Koraga tribe: A study protocol

**DOI:** 10.12688/f1000research.109497.2

**Published:** 2022-12-21

**Authors:** Flavia Sharlet Noronha, Tessy Treesa Jose, Anice George, Linu Sara George

**Affiliations:** 1 Psychiatric (Mental Health) Nursing, Manipal College of Nursing, Manipal Academy of Higher Education, Manipal, Udupi, Karnataka, 576104, India; 2 Pediatric Nursing, Manipal College of Nursing, Manipal Academy of Higher Education, Manipal, Udupi, Karnataka, 576104, India; 3 Fundamentals of Nursing, Manipal College of Nursing, Manipal Academy of Higher Education, Manipal, Udupi, Karnataka, 576104, India

**Keywords:** Koraga, Tribal, Gatekeeper behaviour, mental help, attitude, mental health problems, mental health knowledge, GTP

## Abstract

**Aim:** This study aims to build the capacity of the people at grass root level as gatekeepers of mental health. It will assess the effectiveness of the Gatekeeper Training Program (GTP) on gatekeeper behaviour, awareness, attitude, and mental help seeking intention.

**Design:** An evaluative research approach in two phases. Phase 1: Cross-sectional house-to-house exploratory survey. Phase 2: A quasi-experimental design with multiple follow ups at 0, 6 and 12 months.

**Method:** Data will be collected using standardized tools like Mental Health Knowledge Questionnaire (MHKQ), Community Attitude towards Mentally Ill (CAMIS), Mental Help Seeking Intention (MHSIS) and Gatekeeper Behavior Scale (GBS). For Phase 1, a house-to-house survey will be conducted among the selected colonies of Koraga tribe to determine their awareness, attitude, and mental help seeking intention regarding common mental health problems. Phase 2 includes identification of the leaders/representatives of the selected tribal colonies, and involving them in GTP. Pre-test and multiple post-test will be conducted in Phase 2 at 0, 6, 12 months.  The study is funded by Indian Council of Medical Research from 16 August 2021 for 3 years duration.

**Discussion:** Treatment gap in psychiatric disorders remains an issue of great concern. Evidence based research promotes task shifting approaches in dealing with mental health problems in the community. Capacity building programs like GTP for the underprivileged section of the society are important especially in low and middle income group of countries.

**Impact:** This need based GTP, will ensure mental health first aid in the society. Early identification of people with mental health problems at their doorsteps has huge impact on the prognosis of the illness, closing the treatment gap and stigma reduction.

## Introduction

Tribal communities in India cannot be classified as one homogenous group, as they vary at different levels of development and belong to other ethnic-lingual groups. There are around 42,48,987 tribal people in Karnataka, of whom 50,870 belong to the primitive group. This primitive group of the tribal population represents 6.95 percent of the total population of Karnataka. The Koraga tribe is one of the most backward tribes classified under Particularly Vulnerable Tribal Group (PVTG) as declared by the Government of India. This tribe is scattered over many districts of the State, particularly in Udupi and Dakshina Kannada. Their number is 14,794 as per the 2011 census. The occupation of the Koraga tribe is at the pre-agricultural stage of development, i.e., they are experts in basket weaving, which is the primary source of income, but presently majority of them work as daily wage laborers ( Pujar *et al.*, 2017). Poverty and illiteracy are the main problems in their community ( Roy *et al.*, 2015). They are the poorest among the scheduled tribes of Karnataka ( Nalinam, 2013); around 48.4% of Koraga population have a meager monthly income of Rs.2001-4000 ( Pujar *et al.*, 2017). Alcoholism is another threat to the mental well-being of the Koraga tribal members. Several studies have reported widespread alcoholism among Koraga men and women; they also indulge in smoking beedi (tobacco) and chewing betel leaves. They spend a significant portion of the paltry income, leaving very little for other day-to-day expenses ( Nalinam, 2013). Even after 72 years of Independence and 60 years of the abolition of the untouchability practice, primary health care delivery is still inaccessible to the most vulnerable, primitive population. Unfortunately, people from Koraga tribe are still the “untouchables among the untouchable” ( Balakrishnan & Sharma, 2012).

## Background

In the Toto tribe of the Sub-Himalayan region, the prevalence rate of mental illness was 48.97%, with depression being the highest ( Ghosh *et al.*, 2004). A recent study from the West Godavari district of Andhra Pradesh, predominantly comprising people from scheduled tribes, highlighted that 15% of the present generation of the tribal population was affected by common mental disorders like stress, depression, suicide risk, and anxiety ( Maulik *et al.*, 2017). According to National Health and Family Survey (NHFS) 3, 72% of tribal men aged 15-54 years were addicted to tobacco, and 50% of tribal men were addicted to alcohol ( Ministry of Health and Family Welfare & Ministry of Tribal Affairs, 2013). Psychosocial problems were also reported among high school children of the Mysuru region. About 23.7% of children in the tribal area had anxiety disorders, 1.6% had mood disorders, 3.2% had suicidality, and 2.2% were diagnosed with ADHD ( Pradeep *et al.*, 2018). Koraga, a particularly vulnerable tribal group of Udupi district has shown significant liver dysfunction due to chronic alcoholism among its population, irrespective of its gender. Most of the daily wages were spent on alcohol as evident by majority (75%) of them being alcoholics ( Mungli *et al.*, 2013). Several studies have found that in general there is a vast treatment gap when it comes to the accessibility of mental health services. Although there are several reasons for the same, lack of awareness and stigma towards mental illness and mentally ill people are the most prominent. A cross-sectional survey assessing the effectiveness of community mental health programs in a tribal area of South India revealed that the mean score on awareness regarding mental illness was 5.13±2.27 among its study population ( Yalsangi, 2011). However, contrary to this, the majority (64%) of people from Naga tribe could recognize the presence of a mental health problem in a depicted vignette ( Longkumer & Borooah, 2013). Another study on stigma in mental illness revealed that 38.5% of persons with severe mental illness (PSMI) were found poor, and this poverty was strongly associated with stigma related to mental illness (OR 2.60, 95% CI 1.27 to 5.31), and scheduled tribes (2.39, 1.39 to 4.08). Therefore, it was concluded that females or people from lower castes having severe mental illness were more at risk to be economically poor due to stigma related to mental illness ( Trani *et al.*, 2015).

The Systematic Medical Appraisal, Referral and Treatment (SMART) mental health project of Andhra Pradesh found that 7% of people go to faith healers and religious leaders at the very beginning of their mental illness for treatment ( Maulik *et al.*, 2017). The preference for magico-religious healing and traditional practices to overcome mental health concerns is prevalent in South-Asian countries, evidenced by a qualitative study from Bangladesh on tribal communities ( Rahman *et al.*, 2012). As mentioned earlier, the study poses a challenge to the healthcare delivery system in low-income countries, which mainly caters to the plainland population. A randomized controlled trial across 32 colleges of USA evaluated the effectiveness of the gatekeepers training program and found that trainees' self-perceived knowledge, ability, and confidence to identify students in distress were increased ( Lipson *et al.*, 2014). In the long-term effects of community gatekeeper training, 15 (n=40) participants had helped someone at risk of suicide; also, intentions to help and confidence to identify someone at risk of suicide remained high ( Deane *et al.*, 2006). Though common mental health problems are prevalent among the tribal population, as evident by the research studies mentioned above, very little is done to improve their condition in India. Hence, it is vital to improve the mental health care system for our most primitive and underprivileged tribal people and develop effective mental health care programs at the grassroots level.

**Aims and Objectives:** The objectives of the study are to:

• Determine the awareness, attitude and mental help seeking intention regarding common mental health problems among the tribal population using standardized tools.
• Assess the awareness, attitude, mental-help seeking intention and gatekeeper behavior among the leaders/representatives of Koraga tribe regarding common mental health problems using standardized tools.
• Find out the effectiveness of gatekeeper training program on awareness, attitude, mental help seeking intention and gatekeeper behavior among the leaders/representatives of Koraga tribe regarding common mental health problems.
• Determine the number of vulnerable individuals identified, counselled, referred to a tertiary center as and when needed by the trained gatekeepers of the tribal community at 12 months of follow up.

## Methods

### Research approach and design

The study is based on an evaluative approach to meet its objectives. The study will be done in two phases. Phase 1 will be a cross-sectional house-to-house survey where data will be collected from the Koraga tribe and Phase 2 will have a quasi-experimental design which includes an experimental and a control group. There will be pre-test and post-test for both the groups with multiple follow ups at 0, 6 and 12 months. GTP (intervention) will be carried out only for the experimental group.

### Setting and population

The study will be conducted in Koraga tribal colonies of Udupi district, Karnataka. There are around 360 colonies spread across seven blocks of the Udupi district, and each colony comprises a minimum of 8-10 families. The colonies are geographically distinct in nature. The total Koraga population is 11,133 in Udupi district. Variables under Phase 1 of the study will be awareness, attitude, and mental help seeking intention and Phase 2 variables include awareness, attitude, mental help seeking intention and gatekeeper behavior regarding common mental health problems.

### Sampling technique

Phase 1: Based on convenient sampling technique, out of the seven blocks, three blocks will be taken up for the survey. Each of these three blocks comprises a minimum of 35 colonies, hence 25-27 colonies will be selected randomly from each block. Stratified sampling technique (age and gender) will be used to select five samples/colony for the survey.

**Figure 1.**
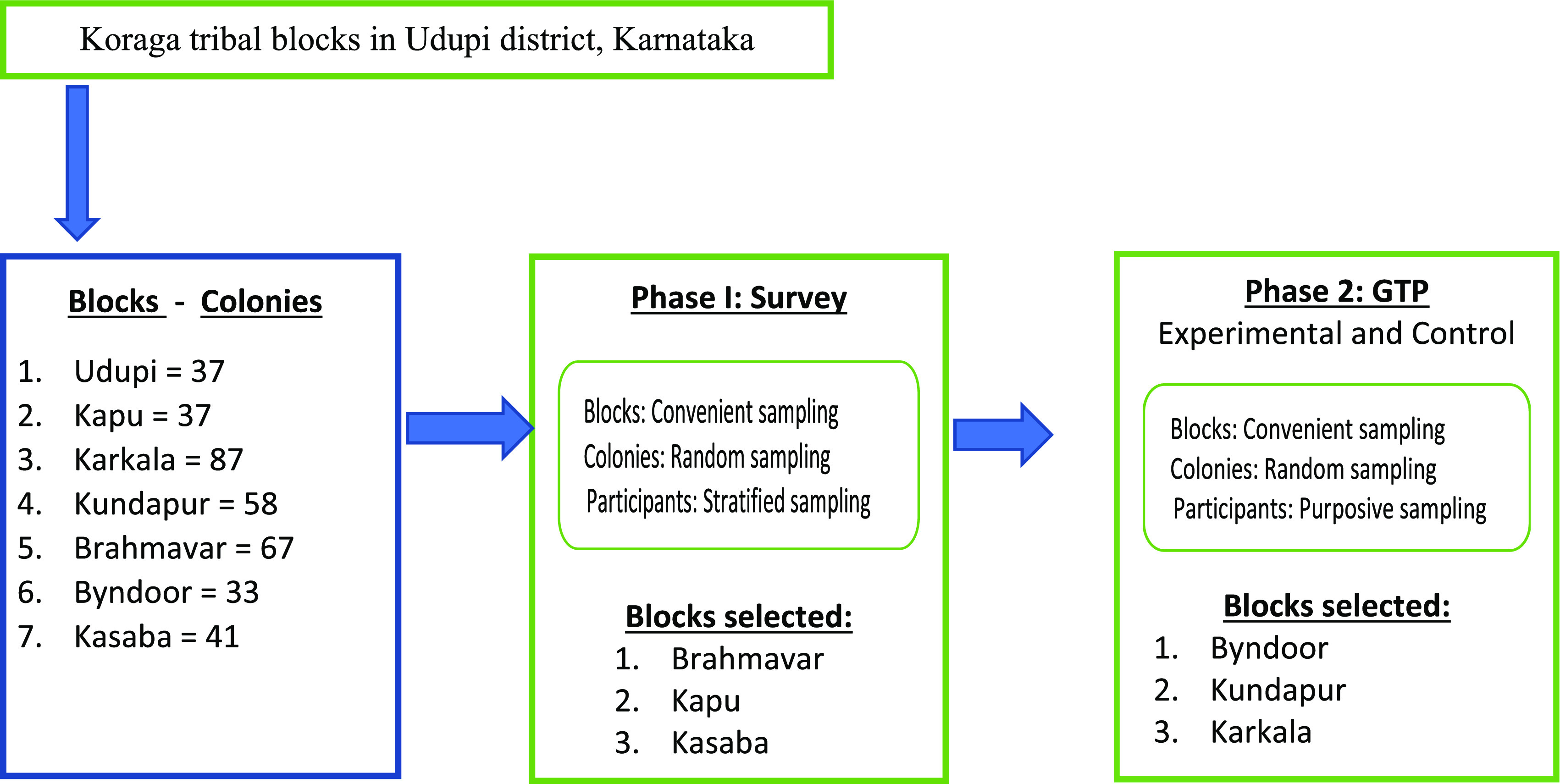
Schematic representation of sampling technique.

For phase 2, convenient sampling technique will be used to select the block (largest block) for experimental and control group. These largest blocks consist of 85-90 colonies, hence 55 colonies will be selected randomly. Further purposive sampling technique will be used to select the participants for GTP ( Figure 1: Schematic representation of sampling technique).

### Sample size

Phase 1 sample size is calculated using the formula as mentioned below. In this formula the confidence level is 95%, hence Z ^2^ = 1.96. Here proportion of the tribal people having awareness regarding common mental health problems was assumed to be 0.5 and (d) is the precision of the study (corresponding to the effect size) = 0.05.

$$n=\frac{Z^{2}P\;\left(1-P\right)}{d^{2}}=384\approx 400$$

For Phase 2: Using G* power software the sample size was estimated to be 86 (43 in each group).

With 20% attrition rate n = 108 (54 in each group).

(Here the values are as follows: Effect size = 0.25, Alpha error = 0.05, power = 0.8, Number of groups = 2, Number of measures = 3.)

### Eligibility criteria

Phase 1: People aged between 18-60 years will be selected in a stratified manner (age and gender) and will be included in the study. For phase 2, (both experimental & control groups) block leaders and representatives from each colony who can read & write Kannada and are willing to participate will be included in the study. The exclusion criteria for both Phase 1 & 2 will be those individuals who have attended previous mental health workshops and those who have any acute/chronic illness.

### Data collection

For Phase 1 data will be collected using standardised tools like Mental Health Knowledge Questionnaire (MHKQ), Community Attitude towards Mentally Ill scale (CAMIS), and Mental Help Seeking Intention scale (MHSIS). Administrative permissions for the data collection is obtained. The data will be collected from the participants after explaining the participant information sheet and obtaining informed consent. Data collection will be carried over six months due to geographical constraints.

For Phase 2, identification of the participants for the GTP i.e the leaders/representatives of the selected tribal colonies will be done with the help of Department of Tribal Welfare, at the district administrative office. The project staff will approach the selected leaders/representatives of the tribal colonies, explain the participant information sheet, obtain consent and then proceed with data collection. Data for pre-test and multiple post-test (at 0, 6, 12 months) will be collected using standarised tools such as MHKQ, CAMIS, MHSIS and Gatekeeper Behavior Scale (GBS). The project staff will be present during the data collection procedure in both phases of the study.

Confidentiality of the data collected: The consent forms will be coded, and any identification data will be concealed thoroughly for both phase 1 and phase 2. The data collection for phase 1 will be done through house-to-house survey using structured questionnaires and data collection for phase 2 of the study will be done during the GTP, that is, the two-day workshop. Structured questionnaires will be used to collect the data. There is no in-depth interviews or verbatim reports involved in this study, however if the participants need any clarifications while filling out the structured tools, the primary investigator will be there to clarify it.

### Data collection instruments

Permission from the authors of the following standardised tools (except socio-demographic) are obtained.

Tool 1: Background information consists of 15 items related to the socio-demographic characteristics of the participants. This tool is developed by the researcher and subjected to validation by experts.

Tool 2: The Mental Health Knowledge Questionnaire is a dichotonoums scale consisting of 25 items regarding substance use disorder, depression, suicide, stress, anxiety, its treatment and prevention. Higher the scores attained on this scale indicates greater mental health literacy. This scale was developed by Hanhui Chen, Zhizhong Wang and Michael Roberts in 2013.

Tool 3: The Community Attitude towards Mentally Ill scale is 40 item Likert scale measuring the attitude towards mental illness and mentally ill people under four dimensions namely authoritarianism, benevolence, social restrictiveness, and community mental health ideology. Higher the score on CAMIS, indicates greater stigma toward mental illness and a mentally ill person. This scale was developed in 1970’s by Martin Taylor and Michael Dear, Canada.

Tool 4: Mental Help Seeking Intention scale (MHSIS) is a 3-item instrument designed to measure respondents’ intention to seek help from a mental health professional if they had a mental health concern with responses ranging from strongly agree (7) to strongly disagree (1). The resulting mean score will range from a minimum of 1 to a maximum of 7. A higher score indicates a greater intention to seek help. This scale is an adapted version from Ajzen’s Theory of Planned Behaviour (2006) and was standardised by Dr Joseph H Hammer and Douglas Spiker in 2018.

Tool 5: Gatekeeper Behavior Scale is a Likert scale consisting of 11 items evaluating the preparedness (5 items), likelihood (2 items), and self-efficacy (4 items) of an individual’s gatekeeping skills. Higher scores indicate better gatekeeping skills of the individual. This scale was developed by Albright, G., Davidson, J., Goldman, R., Shockley, K. & Mitchell-Timmons, J. in 2014.

### Intervention

Gatekepeer Training Program (GTP) is a two-day mental health workshop.

The content for the workshop (module) is being prepared by the primary investigator. Knowledge gap observed from phase 1 (survey) of the study is included in the module. This module will be given to a panel of experts in various fields of psychiatry at local, national, and international level. The suggestions and modifications will be incorporated accordingly.

The program will be implemented by the same multi-disciplinary team members in various community locations as per the convenience of the participants or at a single location if all participants (54) could meet up conveniently.

The investigator will be in contact with the participants throughout the post-intervention period to review/clarify the issues faced by the gatekeepers, and a diary will be provided to the participants in which they will have to document the details of interactions (if they had) with people having mental health concerns. (The sessions for the two-days workshop is listed in Table 1.) The control group in the study will receive a module on the theoretical concepts of common mental health problems and gatekeeper behaviour. For both the experimental and control group, post-test will be conducted at three points of time i.e., at 0, 6, 12 months. After the follow-up duration of one year, a half-day workshop will be conducted for the control group at various community locations (as per the convenience of the participants). This workshop will focus on the theoretical concepts of the module provided earlier.

**Table 1.** Outline of the two-day workshop on GTP.

Sl	Topic	Time	Resource person
**Day 1**			
1	Introduction to Concepts of Mental Health	9:00-10:00am	Principal investigator (Psychiatry nurse)
2	Types of common mental health problems	10:30-12:30am	Principal investigator
3	Early identification and treatment	2:00-3:00pm	Consultant Psychiatrist
4	Strategies in prevention	3:00-4:00pm	Psy. Social Worker
5	Tribal health and welfare	4:00-5:00pm	Representative Dept. of Tribal Welfare Udupi District
**Day 2**			
6	Introduction to QPR (Question, Persuade, Refer) technique	9:00-1:00pm	Principal investigator
7	Importance of Counselling with role plays, case discussion and paired learning	2:00-4:30pm	Consultant Psychiatrist Psy. Social Worker
8	Conclusion	4:30-5:00pm	Principal investigator

Table 1 depicts the sessions along with the resource person that will be included in the two-day GTP workshop during the Phase 2 of the study.

**Validity and reliability:** All the tools (except socio-demographic proforma) that will be used in this study are standardised tools and are validated internationally. Since the study population is local tribal population, the tools are translated into the language known by the tribals (Kannada). Re-translation back to English is done by language experts. Since the primary investigator is fluent and well versed in the local language (Kannada), extensive training was not required for the application of tools in local languages.

Both the English and Kannada versions were subjected to cross cultural validity through experts in the field of psychiatry, psychiatric nursing, psychiatry social workers, and clinical psychologist. These experts have extensive experience in working with the people of Koraga tribe.

The experts agreed that the tools could be used in a tribal population.

Inter-rater and intra-rater reliability is not applicable to this study as only the primary investigator will be recording the data throughout the study period and this study involves only a single trial with follow ups. However, the tools used in this study were tested for reliability and the values are mentioned below: (refer Table 2)

**Table 2.** Reliability testing of the study tools.

Tool	Test done	Reliability
MHKQ (Yes/No)	Kuder-Richardson	0.97
CAMIS (Likert scale)	Cronbach alpha	0.75
MHSIS (Likert scale)	Cronbach alpha	0.89
GBS (Likert scale)	Cronbach alpha	0.79

Table 2 shows the reliability values for the data collection tools of the study.

**Ethical consideration:** Permission to conduct the study is received from the Head of the Institution, Institution Review Committee and Institutional Ethics Committee (322/2020). Permission is also obtained from the tribal community authority (Integrated Tribal Development Office) at the district government office of Udupi. This study is registered in Clinical Trial Registry of India (CTRI/2021/06/034037 [Registered on: 07/06/2021]. Informed consent will be obtained from all participants, and a complete explanation of the investigation will be provided.

Participation in this study is voluntary; the participants may decline to participate at any time, and they need not give any reason for the same, and such withdrawal shall be without penalty. If they withdraw from the study before data collection is completed, the data collected until they indicated withdrawal will be used in the study report. The sample size is calculated by keeping in mind the attrition rate at 20%, hence the sample size will not be affected with the drop-outs.

**Data analysis:** Demographic characteristics of the study participants will be presented in descriptive summary tables. The outcome variables across the follow up period will be analyzed by repeated measures of ANOVA. The categorical variables will undergo bivariate analysis by applying the Chi-square test for categorical variables. A p-value of less than 0.05 will be considered the criteria for statistical significance. Overall SPSS 26 version will be used to analyse the data gathered.

**Dissemination of results:** The results will be disseminated via presentations at appropriate scientific conferences and workshops. The findings will also be published in peer-reviewed journals, professional and institutional repositories etc. The result will be discussed with the Department of Tribal Welfare and other stakeholders for improvement of mental health awareness, accessibility to meantal health care and appropriate utilization of community-based resources.

## Discussion

The health status of the tribal population is in a pitiable condition (“Tribal Health,” n.d.). They continue to suffer due to inequity and inaccessibility in the health care system ( MOHFW&MOTA, 2013). Traditional healers and magico-religious practices are still popular among the tribal population worldwide (Beals *et al.*, 2005). The stigma associated with mental illness is another cause for the treatment gap in psychiatry. Innovative and decentralized community-based approaches should be adopted to reduce barriers in vulnerable populations. World Health Organization has recognized informal community care by peers or community people as an effective and low-cost method of providing mental health services to the vulnerable population ( The Optimal Mix of Services, 2007). Atmiyata, a community-based intervention carried out in Gujarat, India, which provides support to people experiencing mental health issues, found significant improvement in recovery rates and overall quality of life ( ATMIYATA: A Community-Led Intervention in Rural India|Mental Health Innovation Network, n.d.). Nimgaonkar and Menon (2015) in their community-based task-shifting program found that the self-referrals rates increased from 27% to 57%. Also, there was positive growth in knowledge, attitude, and practice about mental health. Hence much more research is required in these areas, and best practices in the community needs to be identified. Community-based interventions incorporating the needs, active participation, socio-cultural beliefs, and resources of its people are reportedly successful.

### Limitations

The desire to commit oneself to social service and help out a person with mental health issues (gatekeeper) vary from person to person and are very subjective in nature. Also in this study the gatekeeper behaviour in the community will be greatly affected by the acceptance of the services despite the stigma, the gatekeepers' belief system, and inter-sectorial coordination among the community stakeholders. Interaction between the gatekeeper and an individual with mental health problems in the community will be collected as a self-report as the researchers cannot reach at the point of time in the community.

## Conclusion

The information obtained on awareness, attitude, and mental-help-seeking intention regarding common mental health problems will help to build the efficacy of Koraga tribe members in terms of gatekeeping behavior. This study believes that community-based interventions are an integral part of mental health services. Programs that help to build the capacity of the people at the grassroot levels will help in early identification, and referral of individuals with signs and symptoms of possible mental health problems, thus reducing the treatment gap, stigma and making mental health services more accessible.

**Study status:**

The researcher is yet to start with the data collection for the study. At present, the tools were validated for cross cultural validity, and pilot phase of the survey is being planned.

## Data availability

### Underlying data

No underlying data is associated with this article.

### Extended data

Extended data available at Figshare repository: The following datasets are available with their doi:

1. Informed Consent for Phase 1. https://doi.org/10.6084/m9.figshare.19114415.v2 ( Noronha *et al.*, 2022a).
2. Informed Consent for Phase 2. https://doi.org/10.6084/m9.figshare.19114409.v1 ( Noronha *et al.*, 2022b).
3. Participant information sheet for Phase 1. https://doi.org/10.6084/m9.figshare.19114430.v1 ( Noronha *et al.*, 2022c).
4. Participant Informant Sheet - Phase II (Experimental group). https://doi.org/10.6084/m9.figshare.19114412.v1 ( Noronha *et al.*, 2022d).
5. Participant Information Sheet - Phase 2 for Control group. https://doi.org/10.6084/m9.figshare.19114421.v1 ( Noronha *et al.*, 2022e).
6. Background information. https://doi.org/10.6084/m9.figshare.19114418.v1 ( Noronha *et al.*, 2022f).

Data are available under the terms of the Creative Commons Zero “No rights reserved” data waiver (CC0 1.0 Public domain dedication).

## Authors contributions

• Flavia Sharlet Noronha: Conceptualization, Methodology, Funding Acquisition, Validation, Writing – Original Draft Preparation
• Treesa Jose: Conceptualization, Methodology, Funding Acquisition, Project Administration, Supervision, Validation, Visualization, Writing – Review & Editing
• Anice George: Conceptualization, Methodology, Writing – Review & Editing
• Linu Sara George: Conceptualization, Methodology, Project Administration, Writing – Review & Editing
