# Supplementary material for: Effectiveness of Gatekeeper Training Program (GTP) on awareness, attitude, mental help seeking intention and gatekeeper behavior among Koraga tribe: A study protocol
Source: F1000Res. 2022 Dec 21;11:291. Originally published 2022 Mar 9. [Version 2] doi: 10.12688/f1000research.109497.2 (PMC9839944; doi:10.12688/f1000research.109497.2)
Supplement: Peer review history

# Peer review: Reviewer response for version 2

**DOI:** 10.5256/f1000research.141996.r158505

**Published:** 2023-01-20

**Authors:** Mohit Varshney

**Affiliations:** 1 Department of Psychiatry, Institute of Liver and Biliary Sciences, New Delhi, Delhi, India

Approved

Is the study design appropriate for the research question?

Yes

Is the rationale for, and objectives of, the study clearly described?

Yes

Are sufficient details of the methods provided to allow replication by others?

Yes

Are the datasets clearly presented in a useable and accessible format?

Yes

Reviewer Expertise:

Mental health; Addictive disorders

I confirm that I have read this submission and believe that I have an appropriate level of expertise to confirm that it is of an acceptable scientific standard.

# Peer review: Reviewer response for version 2

**DOI:** 10.5256/f1000research.141996.r158504

**Published:** 2023-01-13

**Authors:** Omar S. H. Al Omari

**Affiliations:** 1 College of Nursing, Sultan Qaboos University, Seeb, Oman

I am good with the article, and I have no further changes.

Is the study design appropriate for the research question?

Yes

Is the rationale for, and objectives of, the study clearly described?

Yes

Are sufficient details of the methods provided to allow replication by others?

Yes

Are the datasets clearly presented in a useable and accessible format?

No

Reviewer Expertise:

Mental health

I confirm that I have read this submission and believe that I have an appropriate level of expertise to confirm that it is of an acceptable scientific standard.

# Peer review: Reviewer response for version 1

**DOI:** 10.5256/f1000research.121004.r154892

**Published:** 2022-12-01

**Authors:** Omar S. H. Al Omari

**Affiliations:** 1 College of Nursing, Sultan Qaboos University, Seeb, Oman

Dear Authors,

 I have some comments for you to consider before starting your study. 

- (d) representing the effect size and not the margin of the error; please correct it accordingly. Please revisit the formula and ensure the terms that they used. 
- Same issue with the second formula. That is, 0.8 represents the power and not the beta error. Please let the statistician help you with this. 
- I don't know if the researchers used the original English survey or translated version.  If so, please comment on the translation process. 
- I am still trying to understand whether the program's content has been reviewed by a panel of experts or no. If yes, please mention the process. Researchers need to mention the program delivered in one place or different places and to comment on the inter-rater reliability and intra-rater reliability.  
- The reference below will help the researchers in planning their sample size and will acquaint them with helpful information about inter-rater and intra-rater reliability.

Warm Regards,

Is the study design appropriate for the research question?

Yes

Is the rationale for, and objectives of, the study clearly described?

Yes

Are sufficient details of the methods provided to allow replication by others?

Yes

Are the datasets clearly presented in a useable and accessible format?

No

Reviewer Expertise:

Mental health

I confirm that I have read this submission and believe that I have an appropriate level of expertise to confirm that it is of an acceptable scientific standard, however I have significant reservations, as outlined above.

## References

[1] : Nursing Research: Generating and Assessing Evidence for Nursing Practice. 8th Edition. *Wolters Kluwer Health/Lippincott Williams* .

# Response

**Published:** 2022-12-07

**Authors:** Tessy Treesa Jose

**Affiliations:** Manipal College of Nursing, MAHE, India

Dear Dr. Omar S. H. Al Omari

 Thank you for reviewing our work. We will look into the suggestions and work on it and also we appreciate for providing a credible source of reference.

 Regards.

# Response

**Published:** 2022-12-08

**Authors:** Tessy Treesa Jose

**Affiliations:** Manipal College of Nursing, MAHE, India

1. Thank you for the suggestion. The formula is modified accordingly in the updated version of the article.

 2. The tools used in this study are standardized tools available in English language. Since the study population is local tribal population, the tools are translated into the language known by the tribals (Kannada). Re-translation back to English was done by language experts. Since the primary investigator is fluent and well versed in the local language (Kannada), extensive training was not required for the application of tools in local languages. Both the English and Kannada versions were subjected to cross cultural validity through experts in the field of psychiatry, psychiatric nursing, psychiatry social workers, and clinical psychologist. These experts have extensive experience in working with the people of Koraga tribe.

 3. The content for the workshop (module) is being prepared by the primary investigator. Knowledge gap observed from phase 1 (survey) of the study is included in the module. This module will be given to a panel of experts in various fields of psychiatry at local, national, and international level. The suggestions and modifications will be incorporated accordingly.

  The program will be implemented by the same multi-disciplinary team members in various community locations as per the convenience of the participants or at a single location if all participants (54) could meet up conveniently.

 Inter-rater and intra-rater reliability is not applicable to this study as only the primary investigator will be recording the data throughout the study period and this study involves only a single trial with follow ups. However, the tools used in this study were tested for reliability and the values are uploaded in the new version of the article.

# Peer review: Reviewer response for version 1

**DOI:** 10.5256/f1000research.121004.r140324

**Published:** 2022-09-20

**Authors:** Mohit Varshney

**Affiliations:** 1 Department of Psychiatry, Institute of Liver and Biliary Sciences, New Delhi, Delhi, India

This study aims to focus on gatekeeper training and its impact on identifying mental illnesses.

 A pertinent study with rigorous methodology. Can include details of training for application of instruments in local languages in methodology (since most are in English). And how will respondent confidentiality be maintained during the interview? What will happen to those who agree initially to be gatekeepers but than drop out?

Is the study design appropriate for the research question?

Yes

Is the rationale for, and objectives of, the study clearly described?

Yes

Are sufficient details of the methods provided to allow replication by others?

Yes

Are the datasets clearly presented in a useable and accessible format?

Yes

Reviewer Expertise:

Mental health; Addictive disorders

I confirm that I have read this submission and believe that I have an appropriate level of expertise to confirm that it is of an acceptable scientific standard.

# Response

**Published:** 2022-10-13

**Authors:** Tessy Treesa Jose

**Affiliations:** Manipal College of Nursing, MAHE, India

Respected Sir

 Thank you for reviewing our work and providing imperative suggestions. We shall incorporate your suggestions with apt explanation in our methodology of the study.

# Response

**Published:** 2022-12-08

**Authors:** Tessy Treesa Jose

**Affiliations:** Manipal College of Nursing, MAHE, India

**1. Include details of training for application of instruments in local languages in methodology.**

 The tools used in this study are standardized structured questionnaires available in English language. Since the study population is local tribal population, the tools are translated into the language known by the tribals (Kannada). Re-translation back to English is done by language experts. Since the primary investigator is fluent and well versed in the local language (Kannada), extensive training was not required for the application of tools in local languages.

**2. How will respondent confidentiality be maintained during the interview?**

 The consent forms will be coded, and any identification data will be concealed thoroughly for both phase 1 and phase 2. The data collection for phase 1 will be done through house-to-house survey using structured questionnaires and data collection for phase 2 of the study will be done during the GTP, that is, the two-day workshop. Structured questionnaires will be used to collect the data. There is no in-depth interviews or verbatim reports involved in this study, however if the participants need any clarifications while filling out the structured tools, the primary investigator will be there to clarify it. 

**3. What will happen to those who agree initially to be gatekeepers but then drop out?**

 Participation in this study is voluntary; the participants may decline to participate at any time, and they need not give any reason for the same, and such withdrawal shall be without penalty. If they withdraw from the study before data collection is completed, the data collected until they indicated withdrawal will be used in the study report. The sample size is calculated by keeping in mind the attrition rate at 20%, hence the sample size will not be affected
